# Characterization of Hepatitis C Virus Genotypes by Direct Sequencing of HCV 5′UTR Region of Isolates from Saudi Arabia

**DOI:** 10.1371/journal.pone.0103160

**Published:** 2014-08-06

**Authors:** Medhat K. Shier, Mohammad S. El-Wetidy, Hebatallah H. Ali, Mohammad M. Al-Qattan

**Affiliations:** 1 College of Medicine Research Center, King Saud University, Riyadh, Saudi Arabia; 2 Department of Surgery, College of Medicine, King Saud University, Riyadh, Saudi Arabia; Queen Mary Hospital, the University of Hong Kong, Hong Kong

## Abstract

The current study was designed to determine the Hepatitis C Virus (HCV) genotypes in a representative sample of HCV chronically infected patients in Saudi Arabia. All HCV isolates were genotyped by sequencing of the 5′UTR region and newly identified HCV isolates were identified. Specific universal primers targeting 5′UTR region were used for both amplification and sequencing of all isolates that resulted in 244 bp fragment which represent about 80% of 5′UTR region. Most of HCV isolates in this study were genotype 4 (76.4%) where only few isolates were recognized as genotype 1 (19.6%). All results were compared to HCV reference sequences from LOS ALAMOS HCV database, considering only the complete full genomes for the main phylogenetic analysis. Sequences that showed maximum identity (98% –100%) were selected. Most isolates were identical with HCV genotype 4 references. Some isolates were similar to different subtypes of HCV genotypes 4, 1 and 6. Phylogenetic analysis showed resemblance of most isolates to similar ones from the Far East, North America and Egypt. Using sequence Weblogo, Alignment analysis of isolated HCV genotypes 4 and 1 showed 92% and 95.5% nucleotide conservation, respectively. There was no predominant nucleotide in the varied sites, in both genotypes. All isolated sequences were submitted to GenBank database.

## Introduction

Hepatitis C virus (HCV) is estimated to infect 170 million people worldwide. Chronic infection with HCV leads to progressive liver disease ending in liver cirrhosis and hepatocellular carcinoma [1]. HCV has become a major cause of liver cancer and one of the most common indications of liver transplantation [2]. HCV is an enveloped virus that belongs to the genus *Hepacivirus* in the family *Flaviviridae.* The genome consists of 9.5 kilobases of single-stranded, positive-sense RNA that code for at least 10 viral proteins. The polyprotein is composed of structural (C, E1, and E2) and non-structural (p7, NS2, NS3, NS4A, NS4B, NS5A, and NS5B) proteins which are flanked by 5′ and 3′ untranslated regions (UTR) [3], [4]. HCV isolates are classified into at least six major genotypes (genotypes 1–6), whose nucleotide sequences differ by 31–33% [5]. Recently a novel genotype 7 has been described as well [6]. HCV genotypes can be divided further into subtypes which differ by 10–30%. The most common subtypes of different HCV genotypes include 1a, 1b and 1c in genotype 1; 2a, 2b and 2c in genotype 2; 3a, 3b and 3k in genotype 3; 4a in genotype 4; 5a in genotype 5 and 6a, 6b and 6d in genotype 6 [5]. Moreover, distinct isolates from the same subtype may differ in nucleotide sequence by 5–15% [7]. HCV genotype is frequently assigned by phylogenetic analysis of the 5′UTR, core/E1, NS5B, and/or complete genome sequences [5]. Genotype 1a is common in the United States and Northern Europe. Genotype 1b has a worldwide distribution and is often found to be the most common genotype. Genotypes 2a and 2b are also found worldwide and are relatively common in North America, Europe, and Japan [1]. Genotype 3 is found in India, United States, and Europe. Genotype 4a is most common in North Africa and the Middle East. Genotype 6a occurs in Hong Kong and Southeast Asia, while genotypes 5a and 7 are found in South Africa [8], [9], [10] and the Democratic Republic of Congo [6], respectively.

According to the world health organization (WHO) data, the most affected region of the world with HCV, are central and East Asia and North Africa, compared with North American and European countries [11], [12]. In Saudi Arabia, HCV-4 was the most prevalent genotype followed by HCV-1 whereas genotypes 2, 3, 5 and 6 were rarely reported [13]. The diversity of HCV sequences remains a major obstacle for the development of effective vaccines and therapies. Vaccines designed to induce cellular or humoral responses rely on highly conserved regions [14]. HCV diversity is also an important factor in the response to antiviral therapy since genotype 1 and 4 are less responsive to interferon-α (IFN-α) than genotype 2 and 3 [15]. Various HCV genotypes have emerged in different parts of the world. Long-term endemicity in some regions is reflected in the diversity and multiplicity of subtypes. Subtype patterns have been used to trace the origin of genotypes 1 and 2 to West Africa [16], [17], [18] of genotype 4 to Central Africa [19], [20], of genotype 3 to Asia [21] and of genotype 6 to Southeast Asia [22], [23], [24].

The response to HCV treatment is partially dependent on the infecting genotype. Currently, treatment such as pegylated interferon (PEG-IFN-α) and ribavirin can achieve virologic response rates that range from 41 to 80% [25], [26]. Apart from major genotypes, recombinant strains of HCV have been reported in different parts of the world [27], [28], [29], [30]. Recombination may present a significant challenge to the treatment of HCV infection. In addition, recombination may affect diagnosis as many of the current genotyping methods focus on the 5′UTR [31], [32], and most genotyping studies have included only one region such as C/E1 or NS5B; thus, detection of potential recombination events is unlikely due to methodological constrains [5]. Several population-based studies have analyzed the 5′UTR and/or core/E1 genotype versus that of the NS5B region to identify isolates with discordant genotypes that may indicate recombination [33]. The current study was designed to determine the HCV genotypes in a representative isolates of HCV chronically infected patients in Saudi Arabia and isolate novel newly identified HCV subtypes of the commonly isolated genotypes.

## Materials and Methods

### Ethics and Consent Statements

Subjects, including human material or human data, in addition to all written informed consents have been obtained, documented and provided by pathology department, college of medicine, King Saud University, Riyadh, kingdom of Saudi Arabia (KSA). The project and data forms were approved by the Ethics Committee at College of Medicine and King Khalid University Hospital, King Saud University, Riyadh, KSA in compliance with the Helsinki Declaration (http://www.wma.net/en/30publications/10policies/b3/index.html).

### HCV isolates

Sera from 51 HCV-infected patients with chronic hepatitis referred for antiviral therapy were studied. All patients had a positive test for anti-HCV antibodies. Viral titer was determined by the Diagnostic Molecular Biology Unit, Pathology Department, College of Medicine, King Saud University, KSA; using Real Time PCR technique and Cobas Amplicor Instrument (Roche Molecular Diagnostics, California, USA). High viral titers were used in these studies ranged from 7×10^5^ to 11×10^6^ copies/ml.

### HCV RNA extraction

For HCV genotyping studies involving PCR, viral RNA was extracted from 170 µl of serum by QIAamp Viral RNA Mini kit (QIAGEN, Valencia, California, USA). The RNA pellet was resuspended in 60 µl of TE buffer. RNA concentrations were measured in ng/µl using NanoDrop spectrophotometer (ThermoFisher Scientific, Wilmington, Delaware, USA). The extracted RNA yield ranged from ∼35 ng/µl up to ∼125 ng/µl.

### RT-PCR

One step RT-PCR was carried out using QIAGEN one step RT-PCR kit (QIAGEN, Valencia, California, USA) and GeneAmp PCR system 9700 (Applied Biosystems, Foster City, CA, USA). For both reverse transcription and PCR reactions, 5′UTR universal primers: forward KY80 (5-GCA GAA AGC GTC TAG CCA TGG CGT-3) and reverse KY78 (5-CTC GCA AGC ACC CTA TCA GGC AGT-3) were used [34]. HCV PCR products (nucleotides [nt] 9 to 252; 244 bp) were tested on 1.5% agarose gel electrophoresis. Positive PCR products were purified by enzymatic reaction using EXOSAP-IT kit (Affymetrix Inc. USB Products Cleveland, Ohio, USA) that eliminates unincorporated primers and dNTPs.

### Sequencing reaction and genetic analysis

**Figure 1:**
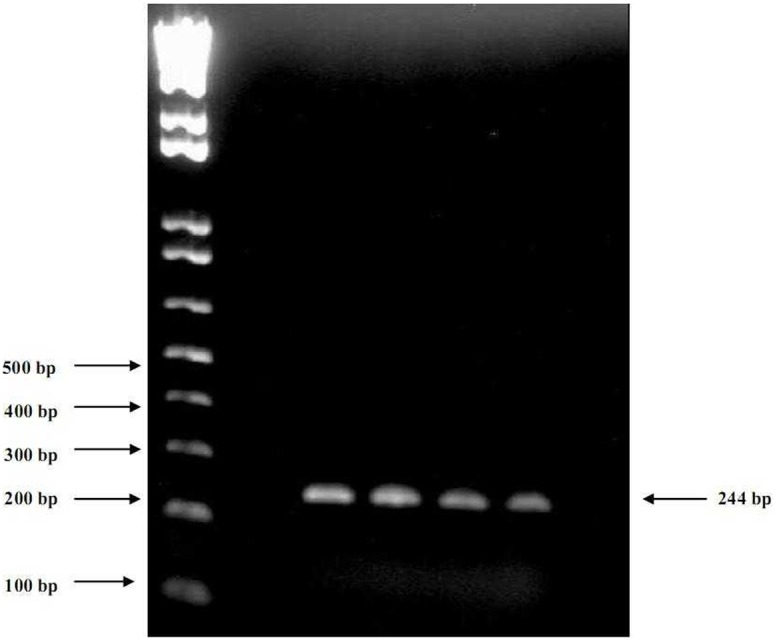
HCV 5′UTR RT-PCR. Viral RNA was extracted from sera of positive HCV Patients and amplified using KY80 and KY78 primer set. Gel electrophoresis was done using 1.5% agarose gel and 1X TBE buffer. The results are shown for the following isolates: 1) EGS201210. 2) EGS201212. 3) EGS201223. 4) EGS201226.

Positive, purified PCR products were used as templates for sequencing in the Big-Dye Terminator V3.1 cycle sequencing kit (Applied Biosystems, Foster City, California, USA) reaction (Figure 1). Isolates were purified from excess incorporated dyes resulted from sequencing reaction using Big-Dye XTerminator purification kit (Applied Biosystems, Foster City, California, USA). The Big-Dye XTerminator purification kit contains XTerminator solution that captures unincorporated dye terminators and free salts from the post cycle-sequencing reaction. The common ethanol/EDTA purification had been used as well. Briefly, 0.1 M EDTA was add to each well then mixed with 100% absolute ethanol, precipitated through high speed centrifugation, then the pellet was cleaned using 70% ethanol, centrifuged and dried by vacuum then samples were dissolved into HI-DI formamide (Applied Biosystems, Foster City, California, USA). Samples were analyzed on an automated sequencer; ABI PRISM 3130 genetic analyzer (Applied Biosystems, Foster City, California, USA). Products were sequenced from both strands to get consensus sequences. The sequence from nt 9 to 252 (244 nt) was taken for analysis. A total of 51 isolates were sequenced in the 5′UTR region.

### Handling raw sequence data

The following steps were used in handling raw sequence date: 1. Collect the sequences for both forward and reverse strands for each samples into separate text file. 2. Add the forward and reverse primers sequences to each corresponding strand respectively. 3. For reverse sequence, get the reverse and complement sequence using the following site: http://www.cellbiol.com/scripts/complement/reverse_complement_sequence.html but keep the forward sequence without any changes 5. Convert all sequences into Fasta format using the following site: http://searchlauncher.bcm.tmc.edu/seq-util/readseq.html 6. Align both forward and reverse sequences into Blast multiple alignment tool using the following site: http://blast.ncbi.nlm.nih.gov/Blast.cgi?PAGE_TYPE=BlastSearch&BLAST_SPEC=blast2seq&LINK_LOC=align2seq 7. Check for mismatching, gaps and short size sequences then re-correct them from each other depending on the QV data from the electropherogram and according to the IUPAC nucleotide code then get the final corrected full size sequence for the sequenced target. 8. To detect the HCV genotype, search nucleotide database using the gotten final sequence in the following site: http://blast.ncbi.nlm.nih.gov/Blast.cgi?PROGRAM=blastn&PAGE_TYPE=BlastSearch & LINK _LOC = blasthome 9. For confirmation, add the final sequence, to the search engine into the HCV database: http://www.hcvdb.org/blast.asp. If the both results from blast the HCV database are corresponding then the sequence is accepted.

### Sequence analysis and phylogenetic tree construction

The new sequences described in this report have been submitted to Gene Bank and can be retrieved under the accession numbers KF999994 to KF999995 and KJ009286 to KJ009313. All isolates sequenced in the present study were aligned with the representative number of sequences for each major genotype and subtype selected from LOS ALAMOS HCV database and Gene Bank using the Multiple Sequence Alignment Program, ClustalW. The original alignment of HCV reference sequences can be downloaded from the HCV database under the subheading “alignments”. Pair-wise comparisons for percent nucleotide homology and evolutionary distance were made. The phylogenetic analysis of HCV isolates was performed with MEGA 5 software. Maximum composite likelihood algorithms were utilized, and phylogenetic trees were constructed by the neighbor-joining method. The reliability of different phylogenetic groupings was evaluated by using the bootstrap re-sampling test from the MEGA program (1,000 bootstrap replications). The following are accession numbers of different HCV reference sequences used in this study; genotype 1: EF032892, AY587016, NC 004102, M67463, EF407419, D14853, AY051292, AF511950; genotype 3: D49374, X76918, FJ407092; genotype 4: FJ462439, FJ025856, FJ025855, HQ537009, HQ537008, FJ462434, FJ462433, EU392173, FJ462440, Y11604, DQ418788, FJ462436, DQ418786, FJ839870, FJ462441, FJ462431, FJ839869; and genotype 6: EU408327, EU408328, DQ835770, EU246931, AY859526, D84263, D84265, EF42625, EU798760, DQ278891, EF424629, D63822, DQ835769, EF632070, EF424626, NC009827, DQ835764, and EU246940.

### Construction of Binding Site Logo

To study sequence variability among various HCV genotypes, the alignment results of 5′UTR sequences were applied into Weblogo software (http://weblogo.berkeley.edu/). Weblogo depicts an alignment as a sequence logo [35], in which each 5′UTR residue is represented as a stack of one letter nucleotide. The height of each stack corresponds to the nucleotide conservation at that position. When the residue is invariant, only one letter is shown, and the most common substitutions are noted when the residue is variable.

### Statistical Methods

Statistical Package for the Social Sciences (SPSS) version 19 software (SPSS Inc., Chicago, IL, USA) was used for statistical analysis. We present frequency and percentages of nominal variables for groups’ genotype 1 and 4 mean, standard deviation (SD) and median for numerical variables (quantitative variables). We used Fisher’s exact test to compare between genotype 4 and genotype 1 with respect to nominal variables. Also, we used non-parametric Mann-Whitney test to compare between genotype 4 and genotype 1 with respect to quantitative variables. We assumed that there was a statistically significant difference when p-value is less 0.05.

## Results and Discussion

A total of 51 patients’ Sera were analyzed. Of the 51 patients, 27 were males and 24 were females (Table 1). The mean age was 49 years (range: 23–88 years). Nationalty of the HCV patients included 49 Saudi (96%), one Palestinian (2%) and one Egyptian (2%). The values of HCV viral load varied from 1,2000 copies/ml to 11,500,000 copies/ml (mean ± standard deviation {SD}: 129,3307±2,649,886), albumin (Alb) varied from 12 g/l to 98 g/l (38±10).Total bilirubin varied from 3 µmol/l to 81 µmol/l (13±12), aspartite aminotransferase (AST) varied from 12 U/l to 175 U/l (41±29), alanine aminotransferase (ALT) varied from 21 U/l to 288 U/l (64±43), alkaline phosphatase varied from 52 U/l to 509 U/l (120±67), and total proteins varied from 42 g/l to 100 g/l (75±8) (Table 1). The only parameter (table 1) that showed significant difference between HCV genotype 4 and 1, was ALT. previous studies have shown that HCV genotype 1 is associated with more severe liver disease and more elevated liver enzymes [36].

**Table 1:** Comparison of HCV genotype 4 and 1 in respect to age, sex, viral load and different liver function tests.

Parameter	Description	Genotype 4 *(n = 39)*	Genotype 1 *(n = 10)*	*P value*
Age	Mean	49.74	47.9	0.851 *
	Median	53	47	
	Std. Deviation	15.096	13.144	
Sex	Male	20 (76.9%)	6 (23.1%)	0.447+
	Female	19 (82.6%)	4 (17.4%)	
Viral load (Copies/ml)	Mean	1468963.58	3912372	0.064*
	Median	599955	2453449.5	
	Std. Deviation	2041281.063	3990716.96	
Tot. Proteins (60–80 g/l)	Mean	75.56	74.75	0.371*
	Median	74.7	78.1	
	Std. Deviation	7.06	12.767	
Albumin (30–50 g/l)	Mean	36.6	42.5	0.874*
	Median	38	36.5	
	Std. Deviation	5.434	20.04	
Tot. Bilirubin (3–17 µmol/l)	Mean	11.24	15.5	0.426*
	Median	10	8	
	Std. Deviation	6.772	23.33	
AST (10–31 U/l)	Mean	39.61	44.4	0.187*
	Median	28	41	
	Std. Deviation	31.396	19.057	
ALT (20–65 U/l)	Mean	62.03	74.3	0.045*
	Median	47	76.5	
	Std. Deviation	47.123	24.18	
Alkaline Phosphatase (50–136 U/l)	Mean	122.16	114.7	0.723*
	Median	103	103.5	
	Std. Deviation	73.565	49.094	

*Mann Whitney test.

+ Fisher’s Exact test.

The HCV genotype distribution in the studied population was as follows: 76.4% (39/51) genotype 4, 19.6% (10/51) genotype 1 and 4% (2/51) unidentified genotype. The two unidentified HCV isolates were giving high percentage of similarity (99% and 94%) with more than one genotype on blast search. Other studies showed that sixty-two percent of Saudis were found to be genotype 4. Other genotypes were 1 (24.1%), 2 (7.4%), 3 (5.9%) and 5 (0.3%). Among non-Saudis (mostly Egyptians), genotype 4 predominated (88%) [36]. In earlier study in KSA, the predominant genotypes among Saudis were type 4 (45.9%), followed by type 1a (40.6%), type 1b (10.8%) and type 2 (2.7%). Type 4 was the major type detected among Egyptians (91.6%), followed by types 1a and 1b (each 4.2%) [37]. In Indian study, the isolates were classified as follows: 53.69% type 3, 38.25% type 1, 6.04% type 4 and 0.671% were type 6 [38]. Saudi patients showed predominance of infection with single subtype of HCV genotype 4.This suggests a recent circulation of a single HCV-4 strain among Saudi patients. Various HCV genotypes have emerged in different parts of the world [5], [6], [39], [40]. Long-term endemicity in some regions is reflected in the diversity and multiplicity of subtypes. Subtype patterns have been used to trace the origin of genotypes 1 and 2 to West Africa [14], [16], [17], of genotype 4 to Central Africa [18], [19], of genotype 3 to Asia [20] and of genotype 6 to Southeast Asia [21], [22], [23].

Phylogenetic analysis of 5′UTR viral region was accurate in determining HCV genotypes while C/E1 and NS5B coding regions were able to differentiate both genotypes and subtypes [41]. The discrimination between major HCV genotypes, which is the strategy commonly adopted in clinical practice, was successfully performed independently of the method used by all laboratories for all isolates except those containing artificially mixed genotypes. However, 5′UTR region genotyping-based methods showed higher sensitivities. In the case of epidemiological studies requiring the precise determination of the HCV subtype, results confirm that NS5B-based genotyping procedures are preferable to 5′UTR region-based ones [42].

### Comparison of HCV isolates with references and the position on the phylogenetic tree

**Figure 2:**
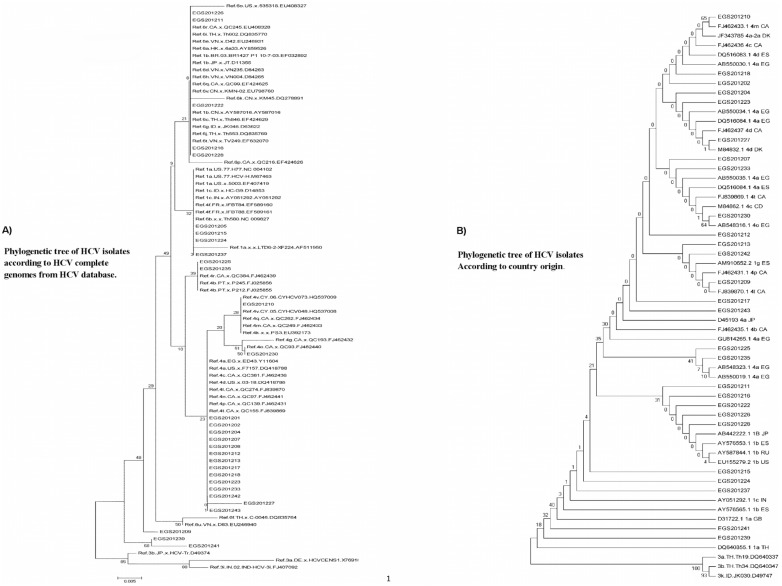
Phylogenetic neighbor-joining tree of HCV 5′UTR partial sequences. Tress were constructed by MEGA V 5.05 software. The numbers of bootstrap replicates supporting relevant nodes are indicated. (A) Sequences for each major subtype were selected from the GenBank database for analysis. The phylogenetic tree was constructed by using the maximum- composite likelihood model and the neighbor-joining statistical method. (B) Sequences from Gene Bank database were selected depending on the regional distribution of different HCV genotypes, picked up from sequence alignment results. The phylogenetic tree was constructed by using the Jukes-Cantor model and the neighbor-joining statistical method.

Reference sequences for different HCV genotypes (including 1, 2, 3, 4, 5 and 6) were used to construct the phylogenetic tree, but genotypes 2 and 5, were not evident on the tree because there were no associated isolates. In constructing the phylogenetic tree (Figure 2a), we decided to use the full genome HCV genotype sequences obtained from HCV database as references. We excluded synthetic sequences, partial or complete CDs. On blast search, isolate EGS201226 gave 99% identity with complete genomes of genotypes 6 and 1b. The identity between these genotypes in the 5′UTR region is around 98% ±1. So the position of this isolate, in the tree, is between genotype 1 and genotype 6 references. Isolates EGS201211 and EGS201222 gave 100% identity with different references of genotype 1b and also gave some results for references of genotype 6. Isolates EGS201216 and EGS201228 gave 99% identity with references of genotype 1b and no or poor matching with genotype 6 references. So the position of these isolates were at the end of higher sub-tree containing some references from genotypes 1 and 6 and above the second sub-tree containing references from genotype 1.

Isolates EGS201205 and EGS201215 were 100% identical with genotype 1a that coincide with their position in the tree; while isolate EGS201224 was only 94% identical with references from genotypes 1 and 4. Because the order of genotypes 4f after genotypes 1a; EGS201224 isolate’s position was at the end of this sub-tree after isolates EGS201205 and EGS201215. By measuring the identity between 5′UTR regions from genotypes 4f and 1a, EGS201224 was 97%.

Isolate EGS201237 was 99% identical with genotype 4a complete genome. The isolate’s position was at the end of the sub-tree compared with the genotype 1a reference that extends with longer branch to confirm that EGS201237 is not genotype 1. Isolates EGS201207 and EGS201208; despite their position between genotype 4 references, were 99% identical with genotype 1g. It was found that the identities between 5′UTR regions of genotypes 1g, 4a and 4p were around 98–99% explaining why these isolates appeared in this position but more specific sub-typing will be obtained from sequencing other regions. For isolates EGS201209, EGS201241 and EGS201239, the position at the end of the tree was the result of their higher identity when compared with complete CDs and partial sequences only and not with the complete genome references. For all other HCV isolates, there was a 100% identity with HCV genotype 4 references so the isolates’ positions in the phylogenetic tree were completely matching with the blast search results. From these results, it is indicated that sequencing of the HCV 5′UTR region will give high precision diagnosis of the HCV genotype, but comparing the results of different HCV subtypes, there is a lack of conclusiveness in determining which subtype is present.

We constructed another phylogenetic tree to correlate our HCV isolates with different sequence references from different countries (Figure 2b). HCV isolate EGS201210 showed 100% identity with reference FJ462433.1 from Canada which is identified as genotype 4m and confirmed from the higher bootstrap value and sisterhood with our isolate. HCV isolate EGS201227 showed 100% identity with reference M84832.1 from Denmark which is identified as genotype 4d. There is high probability that the origin of EGS201227 isolate may be Danish but that would be confirmed only from genotyping of the other regions. For isolate EGS201230, blast search results gave 100% identity with reference AB548316.1 from Egypt which is identified as genotype 4o, indicating that its origin may be Egyptian. Isolate EGS201209 gave 100% identity with reference FJ839870.1 from Canada which is identified as genotype 4l. For isolates EGS201225 and EGS201235, there were 99% identity with two genotype 4a Egyptian sequences, AB548323.1 and AB550019.1 and the phylogenetic tree showed that they are clustered into one branch. The origin of these isolates may also be Egyptian.

**Figure 3:**
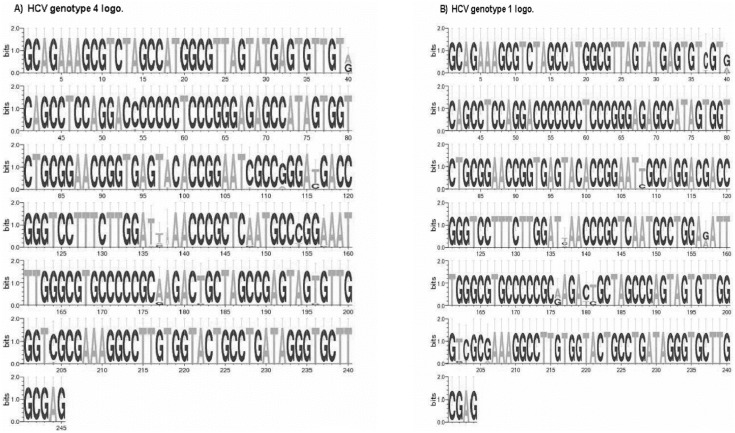
Sequence logo diagram showing the HCV 5′UTR sequences. Numbers shown below the sequence represent the nucleotide position in the HCV complete genome. The alignment was performed by the alignment tool of MEGA 5.05 Software. The logo was build using WebLogo3 software (http://weblogo.threeplusone.com/create.cgi). The vertical bar is 2 bits high. The sites with more than one letter shown per position express the polymorphic sites. The height of each letter represents the relative proportion of each nucleotide at that position. (A) HCV genotype 4 logo using 39 isolates with specific variability depending on bases of single nucleotide mismatches, where all similar sequences were neglected. (B) HCV genotype 1 logo using 10 isolates depending on bases of single nucleotide mismatches.

**Figure 4:**
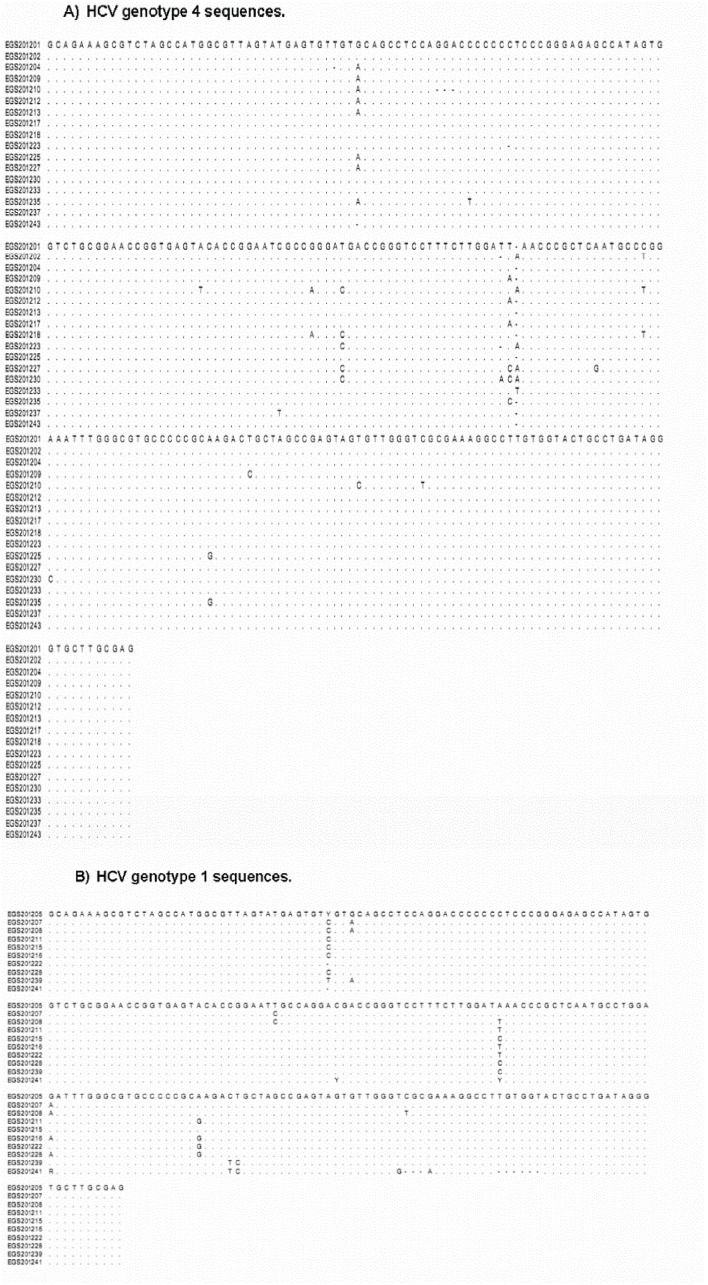
HCV 5′UTR sequence alignment. It shows the mean diversity within the sequences detecting each genotype based on comparison of single nucleotide. Alignment was done using the diversity competition tool of MEGA 5.05 software. (A) HCV genotype 4 using 39 isolates with specific variability depending on bases of single nucleotide mismatches, where all similar sequences were neglected. (B) HCV genotype 1 using 10 isolates depending on bases of single nucleotide mismatches.

Using Weblogo, sequence data of 244 nucleotides were compared in 51 isolates of HCV 5′UTR sequences. For the isolates identified as genotype 4, the logo was created and supported by the alignment analysis (Figure 3a and 4a), 92% (225 nt) of the nucleotides showed conservation among different sequences and only 8% (20 nt) of the whole sequence showed variation. HCV 5′UTR product is 244 nucleotides. However HCV genotype 4 sequence Weblogo (Fig. 3a) showed 245 nucleotides. This can be explained by identifying one gap between position 137 and 138 resulted from the mismatch between different HCV isolates (Fig. 4a). In all cases, gaps of some isolates shifted the amplitude behind the 1.6 bits. There was no predominant nucleotide in the varied sites, but a combination of two nucleotides in one place except in four locations (40, 136, 137 and 138) where the odds are either combination of three nucleotides or absence of any nucleotide to appear as a single gap. For the isolates identified as genotype 1, the logo created and supported by the alignment analysis (Figure 3b and 4b), 95.5% (234 nt) of the nucleotides showed conservation among different sequences and only 4.5% (11 nt) of the whole sequence showed variation. In all cases, gaps in some isolates shifted the amplitude behind the 1.6 bits. A combination of two nucleotides in one place, for some sites, was the mean variation among all sequences.

The diversity of HCV sequences remains a major obstacle for the development of effective vaccines and therapies [39]. HCV diversity is also an important factor in the response to antiviral therapy since genotype 1 and 4 are less responsive to IFN-α than genotype 2 and 3 [40], [2], Previous coalescent approaches used to estimate the epidemic history of HCV, indicate that HCV-4a appears to have been introduced at the beginning of the 20th century and was followed by HCV-4d in the middle of the 20th century [MRCA: 1957 (CI, 1943–1967)] [43], [44], [45]. Phylogenetic analysis revealed two monophyletic clusters (bootstrap value, ∼70) containing HCV infected patients for whom a partial 5′UTR sequence was available. The largest cluster, C1, contained 75% of HCV sequences were identified as genotype 4. Another cluster, C2, contained 30% of HCV sequences located among genotype 1 and genotype 6 HCV references. The vast majority of the patients in C1 cluster were male (96%) and (60%) were Egyptians. Within this so-called Egyptian cluster, phylogenetic analysis revealed also some relativity with references from Canada and Denmark. For C2 sequences, the phylogenetic tree showed these sequences belongs to the area between Japan and USA. In addition to these two clusters, phylogenetic analysis identified 3 unique unrelated isolates all of which were closer to genotype 1a in areas of Thailand, Great Britain and one sequence approaches the 4l strains from Canada. That is similar to findings from previous Saudi study, where the phylogenetic analysis of the 5′UTR showed that Saudi strains (TAIF.SA9, TAIF.SA10) were identical and showed high homology to three 1b strains from Japan (AB049090, D30613 and AF207774) [46]. These findings confirm the global epidemiology of hepatitis C virus infection where HCV genotype 4 is predominant in the Middle East area and genotype 1 that appears in Saudi Arabia and whole gulf area, may be because of large numbers of residents from the Far East and China where these types are predominant [47], [48], [49].
